# Clinical effect of closed reduction minimally invasive fixation in intra-articular comminuted fractures of the femoral condyle

**DOI:** 10.3389/fsurg.2023.1085636

**Published:** 2023-02-03

**Authors:** Xiaodong Lian, Heng Zhang, Fan Guo, Zhongzheng Wang, Kuo Zhao, Zhiyong Hou, Yingze Zhang

**Affiliations:** ^1^ Department of Orthopaedic Surgery, Third Hospital of Hebei Medical University, Shijiazhuang, China; ^2^ Key Laboratory of Biomechanics of Hebei Province, Shijiazhuang, China; ^3^ Orthopaedic Research Institution of Hebei Province, Shijiazhuang, China; ^4^ NHC Key Laboratory of Intelligent Orthopaedic Equipment (The Third Hospital of Hebei Medical University), Shijiazhuang, China; ^5^ Chinese Academy of Engineering, Beijing, China

**Keywords:** femoral intracondylar fractures, minimally invasive, double reverse traction technique, clinical effect, closed reduction

## Abstract

**Objective:**

To evaluate the advantages of double reverse traction closed reduction combined with minimally invasive fixation in treating femoral condylar comminuted fractures.

**Methods:**

We retrospectively enrolled a total of 24 patients with femoral condylar comminuted fractures (AO = 33C3) admitted to Third Hospital of Hebei Medical University from March 2018 to February 2020. The patients were divided into two groups: experimental group (double reverse traction, *n* = 12) and control group (conventional surgery, *n* = 12). Patient demographics, fracture characteristics, operation time, incision length, and postoperative complications were then collected. The Hospital for Special Surgery (HSS) scores were recorded at the last follow-up visit.

**Results:**

The average surgical time was 52.2 (41–73) min in the experimental group and 71.2 (45–103) min in the control group. In addition, the mean total incision length was 13.8 (11–17) cm in the experimental group and 16.3 (14–19) cm in the control group. The average HHS scores at the final follow-up were 86.3 (78–93) and 82.7 (76–90) in the experimental group and control group, respectively.

**Conclusion:**

It was found that double reverse traction closed reduction combined with minimally invasive fixation can provide good repositioning results and functional extremity. Moreover, patients tolerate postoperative functional knee exercises well.

## Introduction

Distal femur fractures account for 0.92% of adult fractures (1). It is worth noting that the intercondylar fracture of the femur is often comminuted and thus it poses a serious challenge to the clinician. The traditional technique requires an incision of the joint capsule and opening of the knee joint, which results in high blood loss during the surgical procedure. In addition, some patients experience severe functional impairment of the knee joint due to postoperative scar adhesions (2). The locking plate screw system for the distal femur provides better results because it can be implanted minimally invasively and with angular stability (3, 4). However, currently, it is only being performed for supracondylar fractures of the femur (AO = 33A), simple femoral condyle fractures (AO = 33B), and two-part intercondylar fractures of the femur without displacement of the articular surface (AO = 33C1, C2). To date, there is no minimally invasive treatment of comminuted displaced fractures of the intercondylar articular surface of the femur (AO = 33C3).

Yingze Zhang's team invented the double reverse traction repositioner (DRTR) technique and used it successfully, with good clinical outcomes, to treat proximal femur fractures, femoral shaft fractures, and tibial plateau fractures (5–7). Herein, we applied DRTR combined with the closed homoeopathic repositioning technique to treat the intercondylar and supracondylar comminuted fractures of the femur from March 2018 to February 2020. Overall, it was evident that the technique minimally invasively fixed the fracture, with good results.

## Patients and methods

This study was approved by the Ethics Committee of Third Hospital of Hebei Medical University (K2015-001-12), and according to the Helsinki Declaration, all patients signed informed consent prior to the study.

### Inclusion and exclusion criteria

The inclusion criteria were as follows: (a) >18 years old; (b) Intercondylar fractures of the femur (AO/OTA type 33C); (c) Closed fractures with less than one month from injury to surgery, or Gustilo type I or II fractures with controlled infection; (d) Patients with no obvious operative contraindication; and (e) Patients who voluntarily agreed to be treated with minimally invasive double-reverse traction surgery and signed the informed consent form.

The exclusion criteria were as follows: (a) supracondylar fractures of the femur (AO/OTA type 33A); (b) simple medial or lateral femoral condyle fracture (AO/OTA type 33B); (c) pathological fractures; (d) old fractures (time between injury and surgery > 1 month); (e) Gustilo type III open fracture, or Gustilo type I or II fracture with uncontrolled infection; and (f) Patients with obvious operative contraindication.

### Patients information

**Figure 1:**
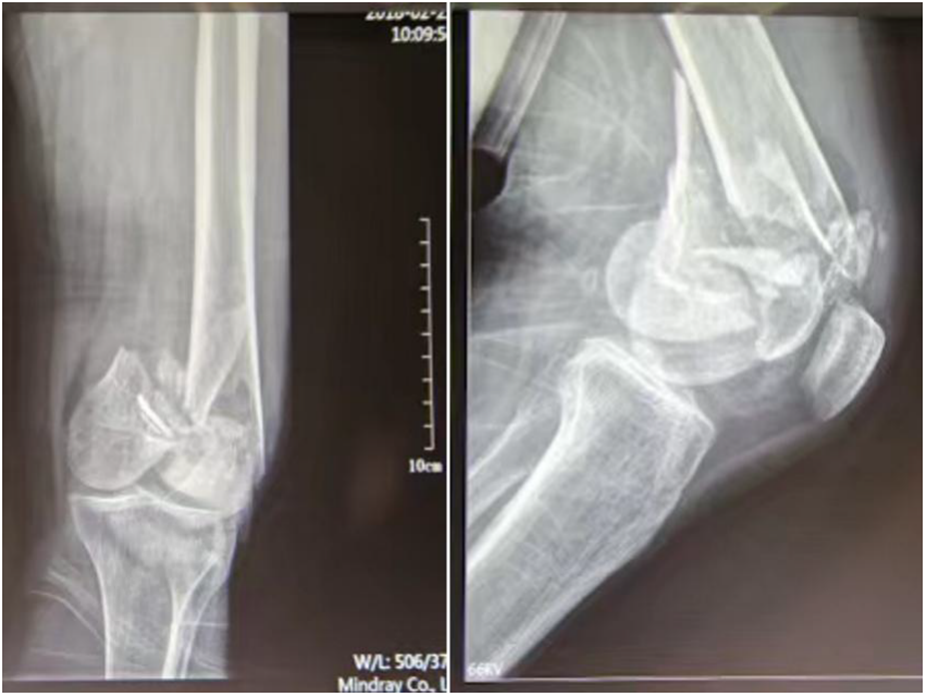
Preoperative x-ray of intercondylar supracondylar fracture of the left femur.

This study retrospectively enrolled 24 patients aged between 32 and 87 years (mean, 53 years). All patients had intercondylar and supracondylar comminuted femur fractures (AO/OTA 33C3) (Figure 1). The mean time from injury to surgery was 8 days (3–19 days). Two patients suffered undisplaced fractures of the tibial plateau on the affected side. Three patients had Gustilo I open fractures, thus, the fractures were only repositioned and fixed after the wounds were closed and free of redness and swelling. One patient was diagnosed with comorbid hemorrhagic shock, which was corrected by blood and fluid transfusion. All participants were divided into two groups: 12 cases in the double reverse traction group (experimental group, A) and 12 patients in the traditional surgery group (control group, B). Demography and fracture-related data for both groups are listed in Table 1, with no statistically significant differences between groups for any of the relevant indicators.

**Table 1:** Demography and fracture-related characteristics.

Patients	Group	Gender	Age (years)	BMI	Injury mechanism	Side of fractures
1	A	Female	68	24.5	Low energy	Left
2	A	Male	58	29.1	High energy	Right
3	A	Male	48	27.6	High energy	Left
4	A	Male	57	24.5	High energy	Right
5	A	Female	64	26.3	Low energy	Left
6	A	Female	46	24.3	High energy	Left
7	A	Male	40	26.4	High energy	Left
8	A	Male	87	20.1	Low energy	Left
9	A	Male	32	25.5	High energy	Left
10	A	Female	37	28.5	High energy	Right
11	A	Male	47	30.4	High energy	Right
12	A	Male	59	26.5	High energy	Left
13	B	Female	67	26.4	Low energy	Left
14	B	Male	52	27.9	High energy	Right
15	B	Male	46	24.6	High energy	Left
16	B	Female	43	26.6	High energy	Left
17	B	Male	36	25.3	High energy	Right
18	B	Male	55	26.8	High energy	Left
19	B	Female	76	21.0	Low energy	Right
20	B	Male	58	28.1	Low energy	Right
21	B	Female	57	24.4	High energy	Left
22	B	Male	35	26.8	High energy	Right
23	B	Female	42	25.3	Low energy	Left
24	B	Male	62	27.6	High energy	Left

### Surgical techniques

**Figure 2:**
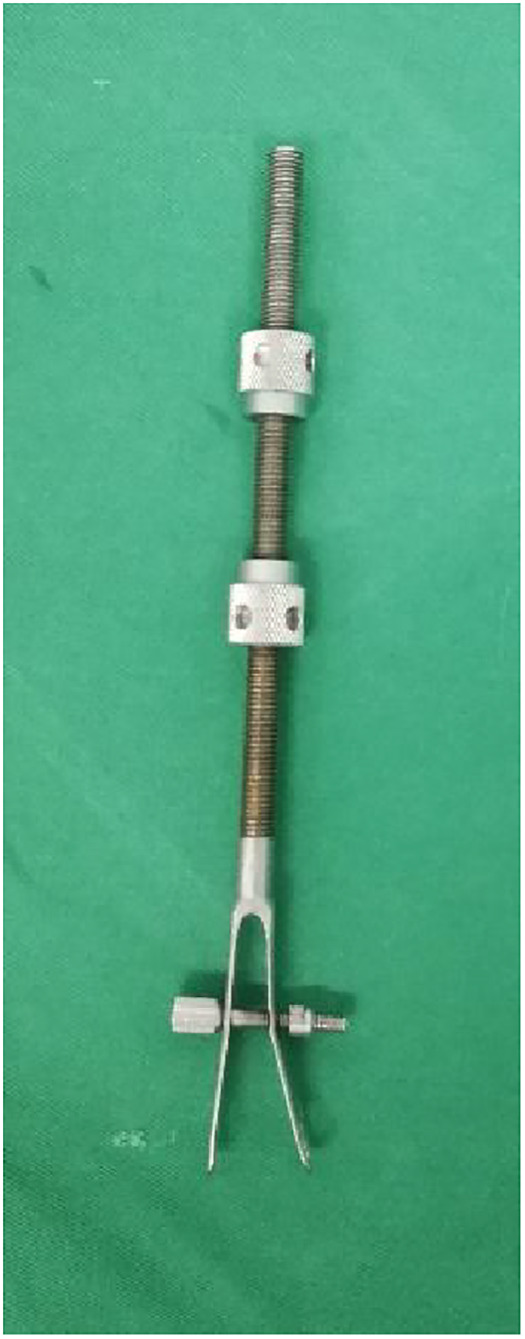
Fork ejector rod of the double-reverse tractor.

**Figure 3:**
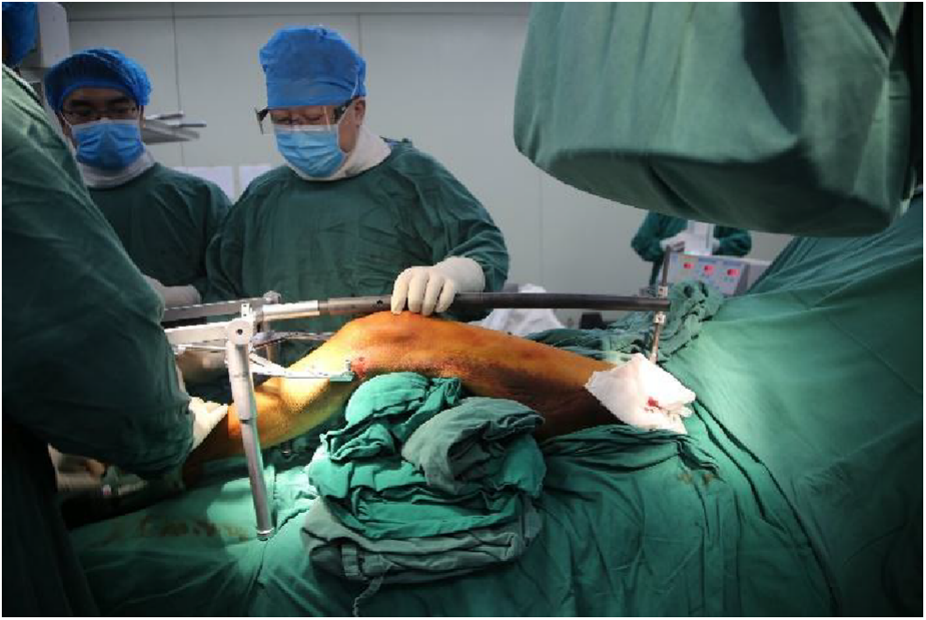
Reduction of fracture with double-reverse traction reducer during operation.

**Figure 4:**
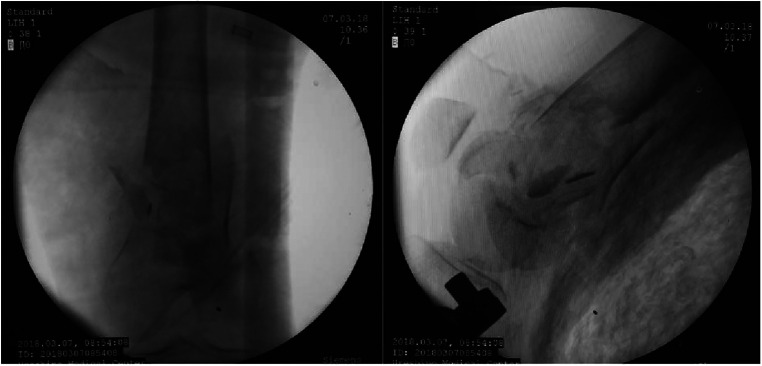
Good fracture position after reduction.

Notably, all surgical procedures were performed by the same team. Surgical approach in the experimental group: After satisfactory general anaesthesia, patients were placed in a supine position. First, two mid-sheet rolls, about 15 cm thick, are placed under the knee joint to keep a flexed position. This relaxes the gastrocnemius muscle and prevents the gastrocnemius muscle from pulling on the femoral condyle, causing the distal fracture fragment to be displaced posteriorly. Subsequently, the fork ejector rod of DRTR was installed 1 cm posterior to anterior superior iliac spine (Figure 2), followed by implanting a 2.5 mm Kirschner wire into the tibial tuberosity to place the tension retractor bow, and then the ejector rod was connected to the tension retractor bow with the frame of DRTR. Appropriate traction was applied by rotating the handle of the DRTR, which repositioned the intercondylar fracture by compression of the surrounding joint capsule, ligaments, and muscles (Figure 3). Next, the femoral supracondylar fracture was repositioned. C-arm fluoroscopy was then used to confirm that the fracture was well repositioned in both anteroposterior and lateral views (Figure 4). If intraoperative fluoroscopy revealed posterior displacement of the distal femoral condylar bone mass in lateral view, tibial tuberosity bone traction would be changed to traction at 1 cm proximal to the articular surface of the femoral condyle. Traction should be positioned appropriately close to the anterior to prevent the gastrocnemius muscle from obstructing the reduction of the femoral condyle fracture.

**Figure 5:**
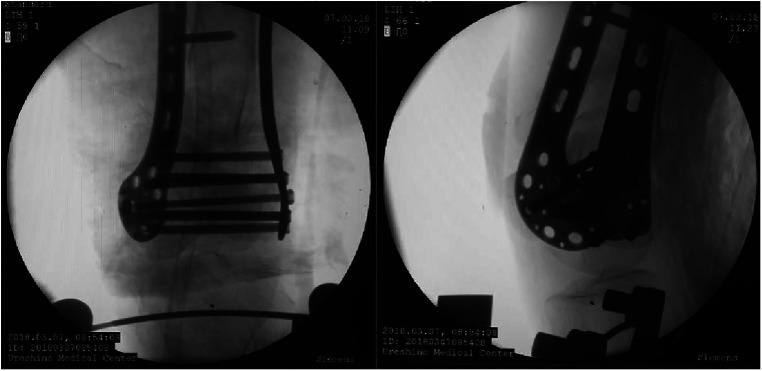
Perspective position after minimally invasive placement of the plate.

After a satisfactory fracture reset, a 3 cm long incision was made on the medial and lateral sides of the distal thigh, followed by placing the medial and lateral anatomical locking plates into the minimally invasive incision percutaneously. Compression fixation of the intercondylar fracture of the femur was then carried out using one compression screw, then screwed in sequence. Finally, the position of the fracture and internal fixation was confirmed by fluoroscopy (Figure 5), and the knee joint was examined intraoperatively to prevent missed knee ligament injuries.

Surgical method of the control group: First, a longitudinal incision was made on the anterolateral aspect of the distal thigh, extending in an arc through the lateral edge of the patella to the lateral tibial tuberosity, with a length of approximately 16 cm. Subsequently, the iliotibial bundle was incised and bluntly separated between vastus lateralis and vastus intermedius muscle. The parapatellar ligament was incised 1 cm lateral to the patella to lift the patella medially to expose the fracture completely. Clean up the soft tissue and blood clots between the fracture ends. The intercondylar fractures were first anatomically reduced under direct vision and fixed with two lag screws. The supracondylar fracture was then reduced by traction on the femoral condyle and temporarily fixed with two Kirschner wires, followed by fixation of the fracture with one locking splint and screws.

### Postoperative management and efficacy assessment

Postoperatively, cefazolin was routinely applied prophylactically. On the first day after surgery, all patients were injected with subcutaneous low-molecular-weight heparin sodium to prevent venous thrombosis. Postoperative anteroposterior and lateral views x-rays of the distal femur showed good alignment and force lines, flat joint surfaces, and well-positioned internal fixation. Patients were encouraged to start foot and ankle activities immediately, and perform knee flexion and extension exercises under analgesia. Anteroposterior and lateral views x-rays of the knee were performed at 1, 3, 6, and 12 months after surgery, and at other time points when necessary. After radiographs showed continuous bone crust growth, patients were encouraged to gradually conduct partial weight-bearing exercise with a walking stick. Notably, full weight-bearing training was only recommended when the fractures healed, and lower limb venous ultrasound was reviewed before patients walked on the floor.

The operative time, incision length, and postoperative complications were recorded for each patient. At the final follow-up, the functional recovery of the affected knee joint was evaluated using the Hospital for Special Surgery (HSS) score: excellent: ≥85 points, good: 70–84, moderate: 60–69, and poor: ≤59.

### Statistical processing

Analysis was performed using IBM SPSS Statistics for Windows, version 19.0 (IBM Corp., Uinted States). Quantitative data were expressed as `*x *± *s*. Surgical time, total length of incision, HSS score were compared between groups using paired *t*-tests. Categorical data was was analyzed by Fisher's exact test. *P* < 0.05 was considered statistically significant.

## Results

All patients had grade A healing of the incision. The length of postoperative follow-up ranged from 12 to 36 months (mean, 17.5 months), no statistically significant difference between groups. Mean surgical time was 52.2 min (range from 41 to 73 min) in the experimental group and 71.2 min (45 to 103 min) in the control group. The mean total incision length was 13.8 cm (range from 11 cm–17 cm) in the experimental group and 16.3 cm (14 cm–19 cm) in the control group. All patients had no varus or valgus deformity of the knee and no knee infections. All fractures healed within 3 to 6 months after surgery (mean of 3.6 months). Results showed that the average HHS at the final follow-up were 86.3 (78–93) and 82.7 (76–90), respectively. Table 2 shows the statistical results.

**Table 2:** Statistical results.

	Experimental group	Control group	*P* value
Surgical time (min)	52.2 ± 15.5	71.2 ± 13.8	<0.05
Total length of incision (cm)	13.8 ± 2.8	16.3 ± 4.2	<0.05
HSS score	86.3 ± 5.2	82.7 ± 4.3	>0.05
Fractures healing time (month)	3.4 ± 0.7	3.8 ± 1.0	>0.05

## Discussion

Intercondylar fractures of the femur are intra-articular fractures that occur in both young and elderly individuals (8). With regard to young patients, the fractures are mainly caused by high-energy injuries, typically due to electric or motor vehicle accidents, and are characterized by comminuted fragments, separate intercondylar of the femur, and widened femoral condyle. On the other hand, elderly patients, primarily women, can sustain a distal femur fracture from a low-energy injury, such as a typical fall due to osteoporosis and reduced bone mass (9). The use of CT allows for a more accurate assessment of the type of intercondylar and supracondylar fractures of the femur. Given the advances in surgical techniques and internal fixation devices, surgical treatment of intercondylar and supracondylar femoral fractures has become the consensus. Treatment aims to restore the joint surface as flat as possible, maintain good fracture alignment and force lines, and avoid knee stiffness with early functional exercise.

It should be noted that the traditional procedure requires a longitudinal incision of vastus lateralis, access to the joint *via* the anterolateral knee capsule, stripping of the periosteum, exposure of the fracture fragments, and repositioning and fixation under direct vision (10). Therefore, this surgery technique has numerous disadvantages, including large incisions, heavy bleeding, sizeable soft tissue damage, and high infection or fracture non-union rates. Traditionally, the force line of the affected limb of the patient with a comminuted fracture is challenging to restore, and postoperative quadriceps adhesions will cause knee stiffness (2). Smith et al. (11) analyzed the literature on 694 distal femoral fractures in 663 patients treated by conventional procedures, and found a 19% incidence of fracture re-displacement, a 6% incidence of delayed or nonunion of the fracture, and a 5% incidence of internal fixation failure.

To address these problems, the present study applied the double reverse traction closed homoeopathic reposition to reduce the intercondylar and supracondylar fractures of the femur. A previous study reported that DRTR allows for bone-to-bone traction with a high traction force (12). It follows the mechanical axis of the lower limb, thereby effectively restoring the force line of the affected lower limb. The intercondylar fracture of the femur is repositioned by pressure compression generated by soft tissue tension (6, 13, 14). Considering that this procedure does not incise the joint capsule, does not expose the joint cavity, and does not disrupt the ligaments and the blood flow to soft tissues around the knee, patients can tolerate postoperative functional knee exercises better, and the likelihood of joint adhesions is minimized.

Our results showed that the technique described here had a HSS score of 86.3, and a mean knee flexion and extension mobility of 105° (95° to 120°), which is consistent with patients treated by conventional methods (15, 16). However, this approach had a smaller total incision length, which aligns with the minimally invasive concept. It is well accepted by patients, and facilitates knee flexion and extension exercises. Meanwhile, the LISS plate, which is more commonly used in clinical practice, can only be applied to extra-articular and non-displaced intra-articular fractures through minimally invasive techniques. Notably, treating displaced intra-articular fractures with the traditional method still requires incision for repositioning (15, 17, 18). Therefore, the method described here extends the indications for minimally invasive treatment with the LISS plate.

During the surgical procedure, it is vital that the origin of the medial and lateral heads of the gastrocnemius muscle is located just posterior to the medial and lateral femoral condyles, and that the direction of femoral condyle displacement is associated with pulling of these tissues. As traction is applied with the lower limb in the extended position, the medial and lateral heads of the gastrocnemius muscle will pull the distal end of the femoral condylar fracture posteriorly. During the procedure, a roll of mid-sheet is placed under the femoral condyles so that the knee is in the flexed position to relax the gastrocnemius muscle, a position that facilitates fracture repositioning. Pulling of the gastrocnemius muscle ensures that tibial tuberosity traction alone can lead to a posterior displacement of the distal fragment of the supracondylar femoral fracture during the double reverse traction procedure. Therefore, the articular surface of the femoral condyle is first repositioned by tibial tuberosity traction. Next, after fluoroscopy shows a good position, the knee joint is positioned with one needle, and a 3 mm Kirschner wire is then drilled into the distal femoral condyle at 1 cm from the articular surface. Notably, this Kirschner wire provides both temporary fixation of the repositioned femoral condylar articular surface and fitting of a traction arch to restore the fracture by traction on the femoral condyle. For sagittal separation and displacement of intercondylar fractures of the femur, the separated intercondylar fracture is fixed by a compression bolt with a self-breaking groove and applying compression. Internal compression of intercondylar fractures of the femur also promotes fracture healing.

This method does not require a traction table, but only a set of DRTR. The overall surgical procedure is simple to learn and has low technical requirements, thereby making it less complicated for orthopaedic surgeons to perform and thus it can be achieved at the primary hospitals. During the procedure, the joint capsule is not opened, and all steps are performed outside the joint. Furthermore, patients can tolerate the post-operative functional exercises well, with reduced pain and knee extension and flexion mobility exceeding 90° one month after surgery.

However, the study had some limitations. First, it was not a prospective study. Second, the sample size was relatively small, and thus the findings may not represent the traits of all intra-articular comminuted fractures of the femoral condyle. Third, given that the follow-up period was short, our results may lack sufficient accuracy in predicting long term outcomes. Fourth, there was a higher number of fluoroscopies compared to the traditional surgical approach. Finally, the anterior superior iliac spine incision also introduces additional surgical trauma.

## Data Availability

The raw data supporting the conclusions of this article will be made available by the authors, without undue reservation.
